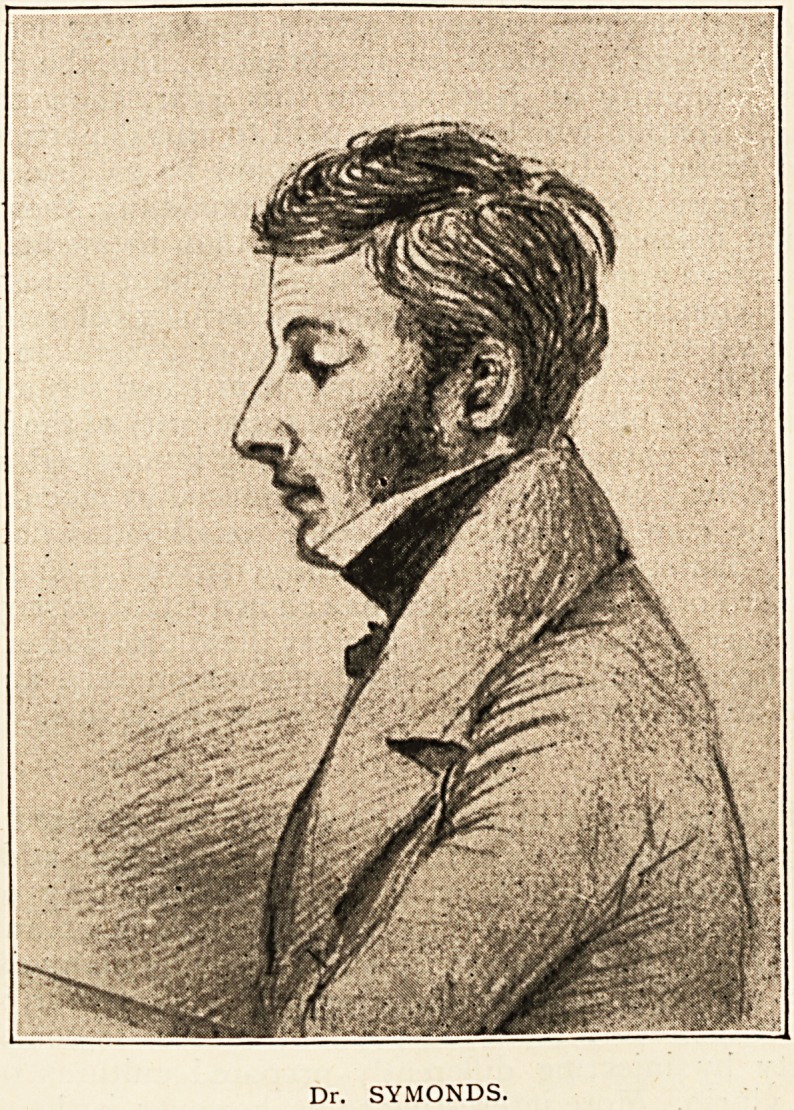# The Early History of the Bristol Medical School

**Published:** 1892-12

**Authors:** Augustin Prichard

**Affiliations:** Consulting Sargeon to the Bristol Royal Infirmary


### THE EARLY HISTORY OF THE BRISTOL MEDICAL SCHOOL.

BY

AUGUSTIN PRICHARD, F.R.C.S. Eng.,

*Consulting Surgeon to the Bristol Royal Infirmary.*

THE formal opening of the handsome new wing of University College, assigned to the Bristol Medical School, and so successfully inaugurated on the 16th of November by Sir Andrew Clark, the distinguished President of the Royal College of Physicians, is not only a fitting occasion for a past history of the school, but seems to demand a record of its work and workers in the earlier part of its life. The following notice, which deals mainly with facts and dates, may be relied on as an accurate account of the school from its first starting into existence in 1832, down to comparatively recent times—that is, for about forty years.

The first prospectus of the Bristol Medical School was issued in 1832, with a preface of some length and somewhat apologetic in tone, setting forth the reasons for establishing provincial medical schools and the advantages of having one in Bristol, which should associate together the several teachers giving independent courses of lectures.

The preface begins thus: "A few years ago it would have been necessary to have supported the announcement of a Provincial Medical School by a series of arguments tending to prove the possibility of obtaining, beyond the walls of the Metropolis, the professional knowledge requisite for the education of students. Happily the necessity for such a demonstration no longer exists;" and stress is laid on the fact that "the whole curriculum of study, both theoretical and practical, prescribed by the Society of Apothecaries[1] may be completed in this city, without repairing to London, except for the purpose of undergoing examination," and that only six months' residence in the Metropolis is necessary before appearing at the College of Surgeons, adding that "the student can have no excuse for neglecting to increase his stores of general knowledge in a place possessed of such valuable sources of information as the Literary and Philosophical Institution, the City Library and the College.[2]" If this was true at that time, we may well ask, What possible excuses for ignorance can be made by any student of the present day with the vastly enlarged facilities for learning which he now possesses? And what increased zeal and energy in teaching must inspire the lecturers who are privileged to lecture with surroundings so convenient, so comfortable, and almost luxurious by comparison!

In the early part of the century many courses of lectures on medical subjects were given in Bristol. In 1814 Dr. J. C. Prichard gave a course on physiology, pathology, and the practice of medicine, at his house in College Green, in which at that time there were no shops. A vine then ran up the front of the house as far as the drawing-room windows. Mr. Bowles and Mr. Shute, two of the surgeons to the Infirmary, and afterwards Dr. Wallis, one of the physicians, gave lectures on anatomy, and held the licence for this purpose; he carried on his dissections in the dead-house of the Infirmary; and later on dissecting rooms were opened by Dr. Riley in Lower College Green, and by Mr. Henry Clark at the back of his house in King Square. The difficulties under which practical anatomy was taught and learned in those days were well known by the exciting narratives of the perilous adven-

[1] The establishment of a fixed curriculum of medical study, and the great improvement in medical education which followed, were principally due to the Society of Apothecaries.

[2] The old Bristol College, in Park Row, where the Synagogue now stands.

tures our immediate predecessors went through. I have seen in the possession of one of these former teachers of anatomy a huge labelled bunch of large keys by which he could have access to any churchyard in Bristol or its immediate neighbourhood. These anatomical lectures were recognised by the authorities in London as early as 1818. In 1816, Mr. Rolfe, formerly Assistant Surgeon to Dr. Thynne, of the Westminster Lying-in Hospital, gave lectures on "The Principles and Practice of Midwifery, illustrated by an ingenious machine and apparatus contrived for the purpose." Mr. Herapath also gave courses upon chemistry.

This is a copy of the first prospectus:

| | TIME. | TERMS. | |
|---|---|---|---|
| | | Single Course. | Unlimited |
| Anatomy and Physiology, by H. Riley, M.D., Physician to St. Peter's Hospital, and Mr. H. Clark, M.R.C.S. | From October to April. | £ s. 4 4 | £ s. 8 8 |
| Principles and Practice of Surgery, by Mr. Hetling, Surgeon to the Bristol Infirmary ... ... ... | December to June. | 3 3 | 5 5 |
| Chemistry, by Mr. Wm. Herapath ... | November to April. | 3 3 | 6 6 |
| Materia Medica and Therapeutics, by Mr. G. D. Fripp, M.R.C.S., Surgeon to the Bristol General Hospital | January to April. | 3 3 | 5 5 |
| Theory and Practice of Medicine, by H. Riley, M.D., &c. ... ... ... | April to June. | 3 3 | 5 5 |
| Midwifery and Diseases of Women and Children, by Mr. J. C. Swayne, M.R.C.S., Senior Consulting Accoucheur to the Bristol Dispensary | January to April. | 3 3 | 5 5 |
| Forensic Medicine, by J. A. Symonds, M.D., Physician to the Bristol General Hospital... ... ... ... Chemical Toxicology, by Mr. Wm. Herapath .. ... ... ... ... | April to June. | 3 3 | 5 5 |
| Anatomical Demonstrations, by H. Riley, M.D., and Mr. H. Clark | November to April. | 3 3 | 6 6 |
| Botany, by Mr. S. Rootsey, F.L.S. ... | April to June. | 3 3 | 5 5 |

The hours for the various lectures will be so arranged as not to interfere with each other.

This first rank of Medical School lecturers has all passed away—one of them, Dr. George Downing Fripp, at a very advanced age, only a few months ago; and it is but right that a few lines should be written about each of these men, who initiated here a work of so much public importance, and made the way comparatively easy for their numerous successors.

Dr. RILEY, who was very like a Frenchman in appearance and manners, received his chief medical education in Paris, where the late Dr. C. J. B. Williams, whose interesting memoirs were published a few years ago, met him in 1825. He was an excellent anatomist and physiologist, fond of comparative anatomy, and a very zealous and hard-working member of the first body of lecturers. He undertook, with Mr. Clark, the two courses of anatomy and physiology, and the anatomical demonstrations, which meant for each of them a lecture every day from October to April; and besides this, Dr. Riley gave the entire course on the theory and practice of medicine. He also gave, independently of the Medical School, several courses of popular lectures on the contents of the Museum of the old Philosophical Institution at the bottom of Park Street, forming a regular series on comparative anatomy; and these lectures were very well attended, the spacious and comfortable theatre of the institution being generally crowded. The men and the women of the present day are, as a rule, no better acquainted with anatomy and physiology than they were in those days, notwithstanding the expanse and advance in educational matters; and yet it is questionable whether a lecturer on such subjects could now collect so large and appreciative an audience. These lectures having been given for the benefit of the institution, resulted in a contribution of £174 to its funds. Dr. Riley was Physician to St. Peter's Hospital, at that time a regular hospital for medical and surgical cases; and the position was to him, as it had been to many others, the stepping-stone towards the coveted appointment of Physician to the Infirmary. He resigned his lectureship at the Medical School in 1846, in consequence of failing health. He married the daughter of Henry Daniel, who was one of the Surgeons to the Infirmary from 1810 to 1836.

Mr. HENRY CLARK, who shares with Dr. Riley the principal credit of establishing our school, and who has been mentioned before as having opened private dissecting rooms near his house, lived for a considerable time at the bottom of King Square. He was of a Bristol family, and a surgeon in ordinary practice: but he had more than the ordinary love for his profession; he desired to improve it, and had a laudable ambition to climb to the top of the tree. He was a good man of business and organiser, and worked hard. He was elected Surgeon to the Infirmary in 1843, and was in one of the early batches of Honorary Fellows of the College of Surgeons shortly after the institution of the Fellowship. Mr. Clark was one of the most strenuous supporters of the Medical School, and had a long connection with it: for he lectured on anatomy and physiology from 1832 to 1839, on descriptive and surgical anatomy (called "anatomical demonstrations" in the earlier prospectuses) from 1832 to 1835, and gave half the surgical course from 1835 to 1860. When he retired from his share in the lectures on anatomy and physiology, Dr. William B. Carpenter became Dr. Riley's colleague, taking the physiological part of the course. Mr. Clark died in 1861 at the comparatively early age of 59, having accomplished much useful and successful work. One of his brothers, the Rev. Joseph Clark, survives him.

Mr. William Hetling gave two courses of lectures upon surgery. He was Surgeon to the Infirmary from 1807 to 1837.

Mr. William Herapath, who undertook the lectureship on chemistry as well as the toxicological part of the course on forensic medicine, was one of the best chemists of his day; his lectures were very interesting and instructive, and fully illustrated by experiments. He was much consulted in cases of suspected poison and for analytical purposes, and gave important evidence in the case of Palmer, the Rugeley poisoner; he discovered arsenic in the stomach of a Mrs. Smith,[1] who had been buried for more than a year in St. Augustine's Churchyard in this city; for which murder Mrs. Burdock, her former landlady, was hanged shortly afterwards. Mr. Herapath made very few, if any, contributions to chemical literature. He was, in addition to his professional work as chemist, a busy public man, a member of the Town Council, an ardent politician, and an extreme Radical.

Dr. George Downing Fripp's first connection with the Bristol Medical School was in the capacity of lecturer on materia medica and therapeutics, which chair he occupied until 1835, when he resigned in favour of Dr. James Bernard and joined Mr. Clark in the surgical lectures until 1840. In 1844 he was chosen lecturer on anatomy and physiology with Dr. Riley, succeeding to the share of that course which had been given by W. B. Carpenter since 1840; and after two

[1] A preparation of this stomach, showing deposits of yellow arsenic on the mucous membrane, was and probably is still to be seen in the Medical School Museum.

years he made a further change, upon Dr. Symonds's retirement from the school in 1846, and became lecturer on the theory and practice of medicine conjointly with Dr. Budd, finally retiring from the School altogether and leaving Bristol in 1849, having taken a share of four different courses of lectures. He was one of the family of that name so well known locally; and like the rest of them, he had a larger share of artistic and musical talent than falls to the lot of most of us. He designed the die which has been used ever since as the seal or arms of the school, representing Æsculapius and Hygeia, with the city arms. At its first formation he was Surgeon to the General Hospital in Guinea Street, and held the office of honorary secretary and treasurer to the school until 1840, when he was replaced by Dr. Carpenter.

Mr. JOHN C. SWAYNE, one of a family that had many medical members, began the lectures on midwifery when the school opened in 1832, giving a three months' course; and he continued until 1850, being assisted from 1837 to 1845 by George Hetling, son of Mr. Hetling who had lectured on surgery, and for five years from 1845 by his son Dr. Joseph G. Swayne, who the year before had given part of the course on descriptive and surgical anatomy, and who succeeding to the entire midwifery chair on his father's retirement in 1850, continues one of the lecturers on the same subject to the present date, that is, for a period of forty-seven years. Mr. Swayne lived at that time in the corner house of St. James's Barton, near St. James's Square, in the house formerly inhabited by Cottle, the poet, who was ridiculed by Lord Byron. Mr. Swayne afterwards moved into Berkeley Square; and before long retired from practice, and went to live in the country, where he died in 1852. He had large experience in the department of medicine which he taught, and was Senior Consulting Accoucheur to the Bristol Dispensary, which meant that he was liable to be called to all the difficult cases by the midwives who had charge of them.

Dr. John Addington Symonds, our first lecturer on forensic medicine, came to Bristol in 1831, and was only in his twenty-fifth year when he joined the Medical School; he was the first Physician to the Bristol General Hospital, which had just been established in two little houses at the end of Guinea Street. The very great probability, if not the certain promise, of this appointment formed one of the principal inducements to Dr. Symonds to settle at Bristol, where he first of all lived in lodgings in College Green. He had for the two or three previous years, immediately after graduating in Edinburgh, been practising at Oxford, assisting his father, a well-known surgeon there. Dr. Symonds gave this course of lectures in the summer season until 1836, when he was succeeded by Dr. William B. Carpenter, one of a family well known in Bristol, where he had served the usual five years' apprenticeship under Mr. Estlin, and had been pupil at the Infirmary, and one in the first list of students of the school, passing the College of Surgeons' examination in 1835. He was the eldest of three brothers of Mary Carpenter, and, like Dr. Riley, he contributed largely to the funds of the Philosophical Institution by his lectures on scientific subjects, chiefly on physiology. Dr. Symonds then undertook, in conjunction with Dr. James Bernard, the two courses of the theory and practice of medicine, and materia medica and therapeutics, giving one-half of each until 1840, when he gave up the latter part of the work, retaining his share of the lectures on medicine with the same colleague, Dr. Bernard, who retired in 1845, making a place for Dr. William Budd. Dr. Symonds himself finally withdrew from his work at the school in 1846, to be succeeded in his half of the course, as has been already noticed, by Dr. G. D. Fripp. Dr. Symonds was a typical physician of what might now perhaps be considered the old school. Skilful in the diagnosis and treatment of disease, he was also sympathetic and careful and kind; he restricted himself conscientiously to medical cases, was courteous to the practitioners whom he met in consultation, dignified in manner, very clever in the general as distinct from the medical management of his patients, and successful. Although not physically strong, he got through a great deal of work; and being an early riser, he found time for writing many excellent and learned treatises and essays on medical and other subjects; and as he had a great and refined taste, and love for the fine arts of all kinds, as well as the style of a highly educated and accomplished man, his works, both medical and others, were always very acceptable and fully appreciated. He died, full of honour, February 25th, 1871, aged 64.

Mr. Samuel Rootsey, Fellow of the Linnæan Society, was the botanical lecturer. He was an excellent botanist, very thoroughly acquainted with the local flora; so that, in the excursions which formed part of the course, he could always lead his class to the place where the plants, some of them very rare, were to be found. He taught according to the old Linnæan system; for the description of the natural orders had not been universally adopted, although a knowledge of them was expected by the examining board of the Apothecaries' Company. I think it is much to be regretted that botany is no longer a necessary subject which students are obliged to attend; for it is one closely allied to medicine. Mr. Rootsey was a good chemist and linguist, and an educated man, but very peculiar, like a philosopher as described in old storybooks. He had at one time a chemist's shop in North Street, and had many of his bottles of tinctures labelled in Greek characters; but as he preferred wandering about the country, botanising and geologising, to the dispensing of medicines, he was perpetually in money difficulties; and imprisonment for debt being the law at the time, he served more than one term at the old gaol on the New Cut, where he was visited by his friends, who found him always cheerful and happy, apparently not recognising his awkward position. On one occasion he told a visitor that he had discovered an easy plan for paying off the national debt, not troubling to think that he ought to pay his own; his plan was to plant the ocean with some floating seaweed which he named, and to grow corn and other profitable plants upon this new soil. He was very fond of queer etymologies and peculiar explanations of his own imagining, and would sometimes wander about the subject of his lecture in an abstracted way, so that his pupils were not always the quiet and orderly set of listeners they ought to have been. He retained the lectureship until 1854, the physiological part of the course being given by

Dr. W. B. Carpenter, Mr. Thwaites (afterwards of Ceylon), and Dr. Brittan in succession; he was followed in the chair by Mr. Etheridge, and died in poverty in Portland Square at a great age.[1]

These were the first lecturers, and the credit of having founded the school and established it on a fairly firm basis—for such it turned out to be, notwithstanding some periods of great difficulty and discouragement—is entirely due to them; but besides these, and a few others whose names have been already mentioned as the immediate successors to the lectureships, there are some who ought to be mentioned specially, and independently of the complete list introduced further on for the sake of future reference.

George Hetling gave the descriptive and surgical anatomy course for two years, and then lectured on midwifery with Mr. Swayne for seven years, and on physiology for one year. He was the son of Mr. Hetling, who gave the first surgical lectures; he practised in Clifton, and was surgeon to the gaol. He was fond of scientific matters; but he rather abruptly turned his back on his profession, and took clerical orders. He is still living and well, at a very advanced age.

John Colthurst, surgeon, a good anatomist and painstaking lecturer, with much skill in dissecting and demonstration and making anatomical preparations, had lectured in Orchard Street for a year or two before joining the Bristol Medical School in 1837; he gave the lectures on descriptive and surgical anatomy for seven years, being joined in 1843 by myself. He retired from practice long ago, and is still living at Chew Magna.

Dr. Kay lectured on forensic medicine from 1837 to 1854.

Thomas Green, Surgeon to the Infirmary, lectured on surgery with Mr. Clark for seven years from 1840. He died in 1878 at the age of 77.

Augustin Prichard, Consulting Surgeon to the Infirmary, began to lecture on descriptive and surgical anatomy in 1843, continuing the course of 140 lectures, with or without a colleague for eleven years, giving up half the course in 1849, and taking half the course on surgery with Mr. H. Clark up to 1861, and the whole course until 1864, when he retired.

Joseph Griffiths Swayne began in 1844 by lecturing on anatomy for one year, and then joined his father as lecturer on midwifery until 1850, since which time he has continued the course; *i.e.*, for forty-seven years. He is Consulting Physician-Accoucheur to the Bristol General Hospital.

[1] In 1811 Mr. Rootsey published a book on Music, and in 1815 a work on Pharmacy.

William Budd, one of the cleverest of the well-known medical family of Devonshire Budds, joined the school as conjoint lecturer with Dr. Symonds in 1845, and continued to occupy the post for ten years. He was Physician to the Infirmary and died at Clevedon in 1879.

Charles Greig, formerly House-Surgeon to the Infirmary, lectured on surgery with Mr. Clark for two years from 1847 to 1849.

Frederick Brittan, Physician to the Infirmary, was connected with the school from 1848 to 1868, and during that time he lectured on physiology, and vegetable physiology as part of the botanical course, on descriptive and surgical anatomy, and on medicine, the latter subject for thirteen years. He died suddenly in London last year.

Crosby Leonard, Surgeon to the Infirmary, lectured on anatomy for ten years from 1854, and then on surgery for seven years, when he resigned his connection with the school. He died in 1879 at the early age of fifty-one.

R. W. Coe, Consulting Surgeon to the General Hospital, lectured on physiology in 1854, on descriptive and surgical anatomy from 1855 to 1864, and on surgery from 1864 to 1879.

Edward Long Fox, Consulting Physician to the Infirmary, lectured on medicine from 1869 to 1875, and the various courses were afterwards carried on by Mr. Etheridge, Mr. Michell Clarke, Dr. Burder, Dr. T. E. Clark, Dr. Henry Fripp, Dr. Marshall, Dr. Leipner, Mr. Lansdown, Dr. Beddoe, Mr. Coomber, Mr. Tibbits, Mr. Atchley, Mr. Steele, and Mr. Board, until about the year 1870.

The subjoined lists, which may serve for future reference, are of much interest as showing the names of the lecturers and the different courses of lectures they gave, with the dates of their appointment and resignation.

**Descriptive and Surgical Anatomy.**

| | |
|---|---|
| Dr. Riley ... ... ... | 1832 - 1835 |
| Mr. H. Clark ... ... | 1832 - 1835 |
| Mr. George Hetling ... | 1835 - 1837 |
| Mr. Jno. Colthurst ... | 1837 - 1844 |
| Mr. Aug. Prichard ... | 1843 - 1854 |
| Mr. J. G. Swayne ... | 1844 - 1845 |
| Mr. S. H. Swayne ... | 1849 - 1851 |
| Mr. J. C. Neild ... ... | 1851 - 1853 |
| Dr. Brittan ... ... ... | 1854 - 1855 |
| Mr. Crosby Leonard... | 1854 - 1864 |
| Mr. R. W. Coe ... ... | 1855 - 1864 |
| Mr. Lansdown ... ... | 1864 - 1872 |
| Mr. T. E. Clark... ... | 1864 - 1868 |
| Mr. Tibbits... ... ... | 1868 - 1872 |

**Theory and Practice of Surgery.**

| | |
|---|---|
| Mr. Wm. Hetling ... | 1832 - 1833 |
| Mr. H. Clark* .. ... | 1835 - 1861 |
| Mr. G. D. Fripp... ... | 1835 - 1840 |
| Mr. Green ... ... ... | 1840 - 1847 |
| Mr. Greig ... ... ... | 1847 - 1849 |
| Mr. Prichard ... ... | 1849 - 1864 |
| Mr. Coe ... ... ... | 1864 - 1878 |
| Mr. Leonard ... ... | 1864 - 1868 |
| Mr. T. E. Clark ... ... | 1868 - 1869 |
| Mr. Leonard ... ... | 1869 - 1872 |

* No course was delivered in 1834.

**General Anatomy and Physiology.**

| | |
|---|---|
| Dr. Riley . ... ... | 1832 - 1847 |
| Mr. H. Clark ... .. | 1832 - 1840 |
| Dr. W. B. Carpenter... | 1840 - 1844 |
| Dr. G. D. Fripp... ... | 1844 - 1847 |
| Mr. Geo. Hetling ... | 1847 - 1848 |
| Dr. Nicholson ... ... | 1847 - 1848 |
| Dr. Brittan ... ... ... | 1848 - 1855 |
| Mr. Coe ... ... ... | 1853 - 1855 |
| Dr. Martyn ... ... ... | 1855 - 1868 |
| Dr. H. Fripp ... ... | 1857 - 1869 |
| Dr. Beddoe ... ... ... | 1868 - 1869 |
| Mr. Atchley... .. ... | 1869 - 1878 |
| Mr. Steele ... ... ... | 1869 - 1872 |

**Midwifery and the Diseases of Women and Children.**

| | |
|---|---|
| Mr. John C. Swayne... | 1832 - 1850 |
| Mr. Geo. Hetling ... | 1837 - 1845 |
| Dr. J. G. Swayne ... | 1845 |

**Forensic Medicine.**

| | |
|---|---|
| Dr. Symonds ... ... | 1832 - 1836 |
| Dr. W. B. Carpenter... | 1836 - 1837 |
| Dr. Kay ... ... ... | 1837 - 1854 |
| Dr. Martyn .. ... ... | 1854 - 1856 |
| Mr. Michell Clarke ... | 1856 - 1863 |
| Dr. Marshall ... ... | 1863 - 1872 |

**Chemical Toxicology.**

| | |
|---|---|
| Mr. Wm. Herapath ... | 1832 - 1867 |

**Chemistry and Practical Chemistry.**

| | |
|---|---|
| Mr. Herapath ... ... | 1832 - 1867 |
| Mr. Coomber ... ... | 1868 |

**Materia Medica and Therapeutics.**

| | |
|---|---|
| Dr. G. D. Fripp... ... | 1832 - 1835 |
| Dr. Jas. Bernard ... | 1835 - 1843 |
| Dr. Symonds ... ... | 1835 - 1840 |
| Mr. Staples... ... ... | 1843 - 1852 |
| Dr. Fairbrother ... ... | 1843 - 1848 |
| Dr. Stanton ... ... | 1852 - 1856 |
| Dr. Burder ... ... ... | 1856 - 1879 |

**Theory and Practice of Medicine.**

| | |
|---|---|
| Dr. Riley ... ... ... | 1832 - 1836 |
| Dr. Symonds ... ... | 1836 - 1846 |
| Dr. J. Bernard ... ... | 1836 - 1845 |
| Dr. Wm. Budd ... ... | 1845 - 1855 |
| Dr. G. D. Fripp... ... | 1846 - 1849 |
| Dr. Stanton ... ... | 1849 - 1856 |
| Dr. Brittan ... ... ... | 1855 - 1869 |
| Dr. Martyn ... ... ... | 1868 - 1876 |
| Dr. Fox ... ... ... | 1869 - 1874 |

**Botany and Vegetable Physiology.**

| | |
|---|---|
| Mr. Rootsey ... ... | 1832 - 1854 |
| Mr. Etheridge ... ... | 1854 - 1857 |
| Mr. T. E. Clark ... | 1857 - 1864 |
| Dr. Leipner ... ... | 1864 - 1892 |

**Vegetable Physiology.**

| | |
|---|---|
| Dr. W. B. Carpenter . | 1840 - 1844 |
| Mr. Thwaites ... ... | 1847 - 1849 |
| Mr. Brittan ... ... ... | 1849 - 1854 |

The following is a list of the Honorary Secretaries to the School for the first forty years of its existence:—

| | |
|---|---|
| Mr. G. D. Fripp ... | 1832 - 1840 |
| Dr. W. B. Carpenter . | 1840 - 1844 |
| Mr. Augustin Prichard | 1844 - 1854 |
| Dr. Stanton ... ... | 1854 - 1856 |
| Dr. J. G. Swayne ... | 1856 - 1858 |
| Dr. H. Fripp ... ... | 1858 - 1861 |
| Dr. Burder ... ... ... | 1861 - 1879 |

At the first opening of the school there was a large number of students, but as time passed on the number varied considerably; at one period the state of affairs was most unsatisfactory and unpromising, as the fees from the entry of the students very barely covered the actual expenses of the place, and those who went through the hard and unprofitable work of bygone days may well congratulate the present more fortunate generation of teachers for having stepped at last on *terra firma*, promising to be a pleasant and successful and permanent abode. In order to render this early account of the school more complete, it will be well to name a few of the more noted among the five hundred pupils who entered during the period under consideration: W. B. Carpenter, before alluded to, Physiologist, Lecturer in Bristol and London; Tyler Smith, who afterwards became a distinguished obstetrician in London; R. M. Bernard, Surgeon to the Infirmary; H. E. Fripp, Lecturer on Physiology; W. C. Coles, well known in the Bombay Presidency; Joseph G. Swayne, Lecturer on Midwifery; Augustin Prichard, Lecturer on Anatomy and Surgery, and Surgeon to the Infirmary; Charles Highett, late Mayor of Bristol; W. Bird Herapath, F.R.S., a clever, hardworking, scientific man, who died early; Nat. Crisp, House Surgeon to the Infirmary; Crosby Leonard, Lecturer and Surgeon to the Infirmary; Henry Marshall, Consulting Surgeon to the General Hospital, and Lecturer on Forensic Medicine; F. P. Lansdown and G. F. Atchley, Surgeons to the Hospital; Berkeley Hill, son of Commissioner Hill, attended the anatomy lectures, and went to London, was Surgeon to University College Hospital, and died at an early age, not long ago; C. Maunder, Surgeon to the London Hospital; Henry Ormerod of Westbury; T. E. Clark, now Reverend; Mortimer Granville, a well-known and successful physician in London; Edwin Coathupe, our popular and efficient Chief Constable; Henry Grace of Kingswood, and his brothers, Alfred of Sodbury, E. M. of Thornbury (Gloucestershire Coroner), W. G. of universal fame, and G. F. (Fred), who died young; E. C. Board, Consulting Surgeon to the Infirmary; C. Steele, late Surgeon to the Infirmary, and Lecturer; Adolph Leipner, Professor at University College, Bristol; R. W. Tibbits, Lecturer and Surgeon to the Infirmary; L. M. Griffiths, Assistant-editor of this *Journal;* F. R. Cross, Ophthalmic Surgeon to the Infirmary; H. Waldo and J. E. Shaw, Physicians to the Infirmary; Lionel Weatherly of Portishead and Bath; and Arthur W. Prichard, Lecturer and Senior Surgeon to the Infirmary; and very many others who do not happen to hold public appointments, whose names rise up to our remembrance, and who are known to be doing us the greatest credit in the different parts of the country where they are practising.

The house in which our old school was domiciled was situated at the bottom of Old Park Hill, shut away by a large gate from the public view, and closely contiguous to an old house then known as De Boudry's School, in Park Row, now the Certified Industrial School, and immediately behind the playground and fives courts of the old Bristol College; a place probably selected because it was obscure and out of the way, for at that period there lingered a very definite feeling among the public, especially among the poor, against dissecting rooms, which feeling had been at its height not many years before, in consequence of the disclosures at the trial of Burke and Hare for murder. Being thus relegated to this quarter away from observation, the Medical School attracted very little notice from the public, or indeed even from the profession; and the staff and committee of the Infirmary,[1] who depended on the existence of the school for their supply of dressers and clerks, turned to it the cold shoulder, and, as far as possible, declined to recognise the Infirmary as a place for education as well as for the cure of disease; and although after awhile some of the faculty found out their mistake, it was not until some of the lecturers had been elected on the Infirmary staff that proper attention was paid to this important subject. There is no doubt but that the sick are much better cared for where a hospital is thrown open for educational purposes than where students are not admitted, and probably no hospital of any size can be really well served for any length of time unless the attendance of pupils is allowed. It is to be hoped that the weighty words of Sir Andrew Clark, in his opening address, will have some salutary effect on the minds of the people of Bristol in this respect; for, as he said, it is of the greatest advantage to a city to have a Medical School in its midst, for the teachers are obliged to work to keep up to all the latest improvements, and the inhabitants have a much larger share of the benefits of their labour than the individuals who form the working staff. A suggestion may here be thrown out, for those among our citizens who may be convinced by what Sir Andrew Clark said, and willing to help in keeping up the school to its present high point of efficiency. They may contribute to the library, or to the funds of the museum, or they may offer prizes for competition, for special or general proficiency, or entrance scholarships, or make an endowment for the school, or some part of it; and there are many who are able and may be willing to assist this important object now that the matter has been thus prominently brought before the citizens, and the public are beginning to understand that the chief advantage is theirs.

[1] At that time the only recognised hospital in Bristol for the education of students.

We must add, however, that in this now despised and old habitation which has been described, very excellent and hard personal work was done. There was a good museum, to which the lecturers contributed, by themselves making and mounting the preparations required for their own lectures; and fifty years ago our students upon going to London were always credited with being first-class practical anatomists, and well up in the subject. May the present and future generations of students improve in skill and knowledge, in proportion as their handsome and convenient new abode excels the ancient home of the school!

Such was the Bristol Medical School of former days; its subsequent history must be written by other hands. The principal start that it made towards the large amount of success it has now attained dates from the foundation of the University College, and the affiliation of the school to it, a step which gave it some importance in the eyes of the public, and enabled the faculty, with the generous help of the medical profession of the city, to raise funds and erect the conspicuous, convenient, and handsome wing of the College, to be known hereafter as the Medical Wing, and appropriated entirely by the school.

There seem to be some slight signs abroad that the people of Bristol are beginning to shake off some of that lethargy in the matter of science, literature, and the arts which has been so conspicuous for the last half century, and to feel some care for things besides trade, commerce, and politics; and in producing this most desirable change, the University College and Medical School must be allowed to have had a considerable share, and will, I hope, receive a corresponding reward in their future progress. . . . . . .

In the evening of the same day when the new school was opened, Sir Andrew Clark presided at the annual Medical School dinner, which had expanded for this occasion to unprecedented size and importance; for besides the usual company, consisting of the Lecturers and the Medical and Surgical Staff of the Infirmary and General Hospital, and the ordinary number of students, the genial Chairman was supported by the Governors of the College, many prominent citizens and distinguished visitors, as well as by many old students of the School. All passed off in a pleasant and satisfactory way, and the inauguration day of the New Medical School may altogether be pronounced a great success. The 16th of November, 1892, when the actual incorporation of the school with the University College took place, will be a memorable and auspicious day for both institutions.

The following is the Toast List at the dinner:

*"The Queen."*

Proposer . . . . . . . . . . . The Chairman.

*"The Prince and Princess of Wales, and the rest of the Royal Family."*

Proposer . . . . . . . . . . . The Chairman.

*"The Bishop and Clergy of all Denominations."*

Proposer . . . . . . . . . . . The Chairman.
Responder . . . . . . . . . The Dean of Bristol.

*"The Mayor and Corporation of Bristol."*

Proposer . . . . . . . . . . . Mr. Lewis Fry.
Responder . . . . . . . . . The Mayor of Bristol.

*"Our Members of Parliament."*

Proposer . . . . . . . . . . Mr. F. R. Cross.

Responders . . . . . . . { Sir Joseph D. Weston, M.P.

Mr. Charles Townsend, M.P. }

*"University College, Bristol, and the Bristol Medical School."*

Proposer . . . . . . . . . . . Mr. F. J. Fry.
Responders . { The Rev. Dr. Percival, Head Master of Rugby.
Professor Lloyd Morgan, Principal of University College.
Dr. E. Markham Skerritt, Dean of the Medical School. }

*"Past and Present Students."*

Proposer . . . . . . . . . . . Mr. J. Greig Smith.

Responders . . . . . . . . { Surgeon-Major Peck.

Dr. Mortimer Granville.

Mr. W. M. Willis. }

*"The Press."*

Proposer . . . . . . . . . Mr. Nelson C. Dobson.
Responder . Mr. Ernest Hart, Editor of the *British Medical Journal.*

*"The Visitors."*

Proposer . . . . . . . . Dr. R. Shingleton Smith.
Responders . { The Rev. M. G. Glazebrook, Head Master of Clifton College.
Mr. R. L. Leighton, Head Master, Bristol Grammar School.
Mr. J. H. Lockley. }

*"The Chairman."*

Proposer . . . . . . . . . . . . Dr. E. Long Fox.
Responder . . . . . . . . . . . . The Chairman.

Through the courtesy of Dr. Markham Skerritt, the Dean of the Medical School, we are enabled to add the names of the lecturers from the dates given by Mr. Prichard on to the present time:—

**Descriptive and Surgical Anatomy.**

| | | |
|---|---|---|
| Mr. E. C. Board | ... | 1872 - 1876 |
| Mr. N. C. Dobson | ... | 1872 - 1878 |
| Dr. H. Waldo | ... ... | 1876 - 1878 |
| Mr. F. R. Cross ... | ... | 1878 - 1887 |
| Mr. W. H. Harsant | ... | 1887 - |

**Theory and Practice of Surgery.**

| | | |
|---|---|---|
| Mr. R. W. Tibbits | ... | 1872 - 1878 |
| Mr. N. C. Dobson | ... | 1878 - |
| Mr. J. Greig Smith | ... | 1888 - |

**Operative Surgery.**

| | | |
|---|---|---|
| Mr. W. P, Keall... | ... | 1878 - 1889 |
| Mr. W. J. Penny | ... | 1889 - 1891 |
| Mr. C. F. Pickering | ... | 1891 - |

**Practical Surgery.**

| | |
|---|---|
| Mr. Arthur W. Prichard | 1878 - |

**General Anatomy and Physiology.**

| | | |
|---|---|---|
| Dr. E. Ludlow | ... ... | 1872 - 1872 |
| Dr. W. H. Spencer | ... | 1872 - 1874 |
| Dr. R. Shingleton Smith | | 1874 - 1887 |
| Mr. G. Munro Smith... | | 1887 - |
| Dr. J. Michell Clarke... | | 1887 - 1892 |

**Practical Physiology.**

| | | |
|---|---|---|
| Mr. G. F. Atchley | ... | 1878 - 1892 |
| Dr. J. Michell Clarke... | | 1892 - |

**Theory and Practice of Medicine.**

| | | |
|---|---|---|
| Dr. W. H. Spencer | ... | 1874 - 1888 |
| Dr. E. Markham Skerritt | | 1876 - |
| Dr. R. Shingleton Smith | | 1888 - |

**Pathology and Morbid Anatomy.**

| | | |
|---|---|---|
| Dr. R. Shingleton Smith | | 1887 - 1888 |
| Dr. Barclay J. Baron .. | | 1888 - |

**Practical Pathology and Morbid Anatomy.**

| | |
|---|---|
| Dr. Barclay J. Baron .. | 1887 - |

**Forensic Medicine.**

| | | |
|---|---|---|
| Mr. W. P. Keall... | ... | 1872 - 1878 |
| Dr. R. Eager | ... ... | 1879 - |
| Mr. W. W. Stoddart ... | | 1879 - 1880 |
| Dr. A. J. Harrison | ... | 1881 - |

**Materia Medica and Therapeutics.**

| | | |
|---|---|---|
| Dr. J. E. Shaw | .. ... | 1879 - 1888 |
| Dr. A. B. Prowse | ... | 1888 - |

**Midwifery and Diseases of Women.**

| | |
|---|---|
| Dr. A. E. Aust Lawrence | 1879 - |

**Public Health.**

| | | |
|---|---|---|
| Mr. D. Davies | ... ... | 1879 - 1886 |
| Dr. D. S. Davies | ... | 1886 - |

**Comparative Anatomy.**

| | | |
|---|---|---|
| Mr. W. J. Sollas... | ... | 1879 - 1884 |
| Prof. C. Lloyd Morgan | | 1884 - |

**Biology.**

| | | |
|---|---|---|
| Dr. Leipner... | ... ... | 1892 - |
| Prof. C. Lloyd Morgan | | 1892 - |

Dr. E. Markham Skerritt, who succeeded Dr. Burder as Honorary Secretary in 1879, was appointed Dean of the School in 1883, when the office of Secretary was abolished.

Mr. F. Bligh Bond, the architect of the new Medical School, has been good enough to supply us with some architectural notes concerning it[1] :—The new building, standing at

[1] The illustrations are taken from photographs by Mr. Edwin Gael, 77 White Ladies Road, Bristol.

The first view does not give the building quite to the roadway, on account of the height of the hedge of the Grammar School playground from which the photograph was taken. The second view represents not only the front of the building, but also the sides of the Library and Museum.

the extreme lower angle of the University grounds, will, when the whole scheme is completed, form a wing of the great front, detached from the main block by a narrow space only, and square with the rest. The front is for that purpose broken back in a series of parallel faces, an arrangement necessitated by the curve in the road.

The previously existing portions of the University College permanent building were erected from 1879 to 1883 under the superintendence of the late Mr. Charles Hansom, with whom Mr. Bond was in partnership until his death in 1888; but, being intended for back portions only (*i.e.* the back and one side of the future quadrangle), they do not show to great advantage, being somewhat severe in their outline.

The new building has received a careful architectural treatment, befitting its more conspicuous position. In it are reproduced some of the more essential features of the style adopted by Mr. Hansom, with certain modifications of detail that will probably be considered advantageous.

The new School is built in local stone of a warm purplish-red colour, the dressings and architectural features being of Combe Down freestone.[1] The portion containing the large Museum and Library is made to dominate the whole design, and has an imposing gabled front, flanked by turrets with small crocketed cupolas, the Library windows having arched tracery heads of rich pattern. All the remaining windows of the building are square in outline, mullioned, and having small cusped arches in the heads. A fine, deeply-moulded cornice runs round the entire building, broken at intervals by

[1] The contractor's work has been well carried out by Mr. George Humphreys, of Stapleton Road, Bristol.

### NEW MEDICAL WING, UNIVERSITY COLLEGE, BRISTOL.

KEY.

A MUSEUM

B. LABORATORY

C. CLOAK ROOM

D. READING ROOM

E. FACULTY ROOM

F. STUDENTS' ROOM

G. LECTURE ROOM

H. ANTE ROOM

I. LECTURE ROOM

J. MEDICAL TUTOR'S ROOM

K. LATRINES

L. LIBRARY

M. LECTURERS' ROOM

N. PHYSIOLOGY LABORATORY

O. ,, THEATRE

P. ANATOMY THEATRE

Q. PROSECTOR'S ROOM

R. ,, ,,

S. DISSECTING ROOM

T. LAVATORY AND W.C.

GROUND PLAN.

FIRST FLOOR PLAN.

SCALE 10 0 10 20 30 40 50 OF FEET.

carved gurgoyles, pateræ, and grotesque heads. The walls finish with a battlemented parapet.

One enters from the road, under a moulded archway through a little lobby enclosed with pitch-pine screen-work, and paved with mosaics, to find oneself in a spacious hall, floored in a similar manner, whence access may be had to the rooms for Faculty meetings and for students on the left hand, and to a doctors' reading room, a cloak room, and a porter's room on the right. At the end of the corridor on the right hand a doorway, in a screen fitted with fine lead glazing, conducts to the Museum, a well-lit room, 50 feet by 30 feet, with a preparation room in connection with it.

There is a handsome piece of glass by Bell and Son in the staircase lights, which gives the whole staircase a good effect. The design of the balusters and the solid newels is in keeping with the rest of the building, and the walls are cased here, as in most of the rooms, hall, and corridors, with panelled dados of choice figured pitch-pine.

Ascending the broad, old-fashioned staircase which rises from the hall, we arrive at the first floor, where a theatre is provided for physiological lectures, a Laboratory properly fitted for physiological work, a small preparation room, and a Library.

The Library is entered under a richly-moulded arch, with carved spandrels, emblematical of the mysterious principles of life and the healing attributes of the Deity. The room, which is planned and fitted to take the associated libraries of the Medical School and the Medico-Chirurgical Society, is a very handsome one, 50 feet by 30 feet, lit on three sides by mullioned and traceried windows, and dignified by an open timber roof, with panelled compartments, having carved bosses at their intersections, and supported by massive moulded hammer beams, with carved braces and fretted spandrels. These beams rest upon six moulded freestone corbels, bearing shields, on which are cut monograms of initials of some of those who have had a distinguished connection with the School.[1] A handsome freestone chimney-piece, of the old baronial type, stands at one end of the room, and the solid ranges of dark-wood bookshelves complete the circuit of the room. A pleasant effect for reading purposes is produced in this Library by the introduction of cathedral glass in all the lights, a pale green tint slightly predominating.

At the rear of the building a glazed covered way gives immediate access to the old School, which, with a few necessary internal modifications, has been preserved intact. The Lavatories, which are situated at the end of this covered way, are very complete, well lit, ventilated, and drained.

The whole of the new building is lit with gas, the best rooms having powerful sun-burners, with ventilating trunks connected to them, and the heating throughout is by low-pressure hot water.[2] Radiating coils are provided in nearly every room, and wherever else needed, through which continually enters an ample supply of fresh air, warmed to an agreeable temperature.

Mr. J. Greig Smith, who has taken considerable interest in all the work of the new school buildings, and has devoted much care to the details of arrangement and decoration, has very kindly, in response to our request, sent us the following note: "Symbols connected with the healing art are here and there introduced. On a scroll over the front door, and in the Greek characters adopted by the College of Physicians of London, is the well-known Hippocratic aphorism relating to the shortness of life and the length of art: at each end of the scroll is introduced in full relief a bold treatment of the Æsculapian rod and staff. Over the archway that leads to the library are represented various symbols, such as the pentacle introducing at its angles the letters Υ Γ Ε Ι Α; the tree of life; the serpent, with rod and with cup."

[1] These are Dr. Henry Riley, Mr. Henry Clark, Mr. William Herapath, Dr. John Addington Symonds, Dr. Joseph Griffiths Swayne, and Dr. William Budd.

[2] These arrangements have been admirably carried out by Messrs. James Crispin and Sons, of Nelson Street, Bristol.

Dr. J. G. SWAYNE writes: "As just at present everything in connection with the history of the Medical School is of interest, I am sending you some portraits, which are copies of pencil drawings made by me in my note-book in 1840, when I was a student attending lectures and ought perhaps to have been better employed. They represent six of the founders and first lecturers of the Medical School in the Old Park, Bristol, in the year 1832. I regret that I did not in 1840

Mr. HENRY CLARK.

take likenesses of all the first lecturers; but one of them, Wm. Hetling, died before I became a student, and the other two I did not take: for I little thought in 1840 before the days of photography that these drawings might become useful in 1892, after fifty-two years had passed. I add a few notes about them, but must refer the reader to Mr. Augustin Prichard's paper on the Medical School for fuller information."

As a lecturer especially on anatomy Mr. HENRY CLARK was very clear and impressive, and readily imparted his information to his pupils. He was also a good surgeon, though a rather nervous operator. Mr. Henry Clark was succeeded in practice by his nephew, Mr. Thomas Edward Clark, who became a lecturer at the school, Surgeon to the Infirmary, Physician to the General Hospital, and then gave up the profession, and took orders in the Church of England.

In medicine Dr. Henry Riley was an ardent disciple of Broussais, and carried antiphlogistic treatment very far. I

Dr. HENRY RILEY.

remember when I was the pupil for the week at the Infirmary, I once bled and cupped under his directions nearly fifty patients in one day. His diagnosis was good, especially in chest diseases. His clinical remarks were instructive, and he had a good following of pupils in the wards. Like Beau Brummel, he never failed in his tie. Deep and so well starched that it was without crease or wrinkle, it was a fine example of the "white choker."

Besides being a distinguished chemist, Mr. Herapath was a town counciller and a magistrate of some repute. He had a rather quick temper. One snowy morning in winter he had just retired after his chemical lecture, and was walking down the Medical School yard, when some parting shots in the shape of snowballs were fired at him from one of the Medical School windows in the rear. One of these knocked off his hat and quite upset his equanimity. He at once faced round and said: "If I knew who threw that snowball, I'd sink the magistrate, I'd sink the lecturer, and give him as good a

Mr. HERAPATH.

thrashing as ever he had in his life!" This little episode rather increased the medical students' respect for him; for they regarded him as a man who was ready to fight if necessary. He died in 1868, having lectured for 36 years. His son, Dr. William Bird Herapath, was for some years engaged in extensive general practice in Bristol; but, notwithstanding this, he was, as a chemist, more distinguished than his father, owing to his becoming F.R.S. in consequence of a very able paper of his on the polarising properties of the salts of quinine. Dr. Bird Herapath's son is a medical man now practising in Bristol.

My father, Mr. John Champeny Swayne, was at times a very absent man, especially when he was lecturing and fully absorbed in his subject. Some students, aware of this peculiarity, prepared a lay figure, by means of a mop, a mask, and some old clothes, and seated it on a bench amongst them a few yards in front of where he stood to

Mr. JOHN CHAMPENY SWAYNE.

lecture, just to see if he would notice it. He lectured in winter in the afternoon, when the light was not good, and for ten minutes or so, as they expected, he did not notice the figure. However, when he did and fixed his eyes upon it, there was a general explosion. As soon as it was over, my father turned round quietly to the porter, who was registering the absentees, and said: "I 'll trouble you to remove that strange gentleman; but permit me to remark that I wish all the others attending this lecture were as quiet and orderly!"

Dr. Tyler Smith (a fellow student of mine at the Bristol School, who afterwards practised in London, and was appointed Obstetric Physician to St. Mary's Hospital when it was opened) told me that my father's lectures induced him to take up obstetric practice.

Dr. JAMES BERNARD was not one of the original founders of the school, but was one of its earliest recruits. He lectured for ten years; viz., from 1835 to 1845, when he retired. He

Dr. JAMES BERNARD.

was lecturing on materia medica when I was a pupil; his lectures were scholarly and showed much erudition; but the subject was a dry one, and his delivery and manner were not attractive. He soon afterwards was elected Physician to the Infirmary, and resigned his lectureship. In private practice he did well, and was universally respected.

About the middle of this century Dr. SYMONDS was perhaps the most successful physician in the West of England.

He lived in Clifton Hill House, a mansion bearing the date 1747, and built at a time when Clifton on the hill was an outlying village. Dr. Churchill, the eminent accoucheur of Dublin, once remarked to him, in my presence: "Doctor, you have the finest physician's house I have seen anywhere." Before Dr. Symonds bought it, it was occupied by Mr. George E. Sanders, a gentleman of fortune, and a leading Liberal. At that time, in the forties, the two hospitals were highly political, just as the Colston dinners are at the present

Dr. SYMONDS.

time. The General Hospital, then a young struggling institution, was chiefly supported by the "Anchorites;" whilst the "Dolphinites" clave to the old-established "Charity Universal," Bristol Infirmary. Permission had just been granted to call it the Bristol Royal Infirmary, whereupon Mr. Sanders remarked: "The patients who want a Sovereign remedy will now go to the Royal Infirmary; but those who want a Radical cure will go to the Hospital!"

## Figures and Tables

**Figure f1:**
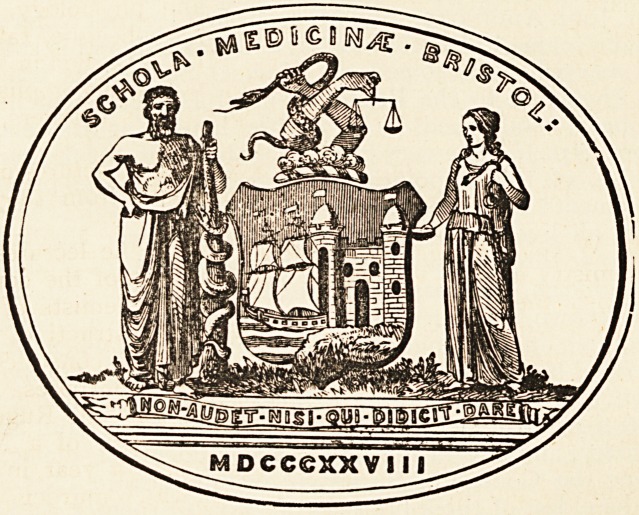


**Figure f2:**
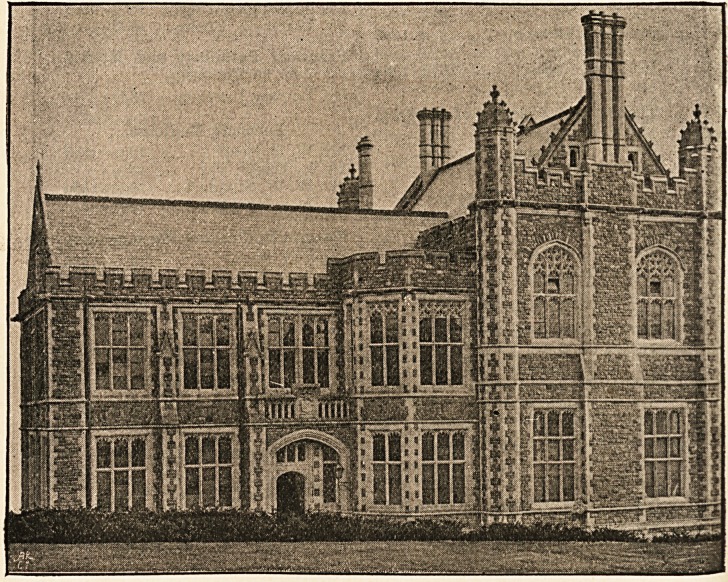


**Figure f3:**
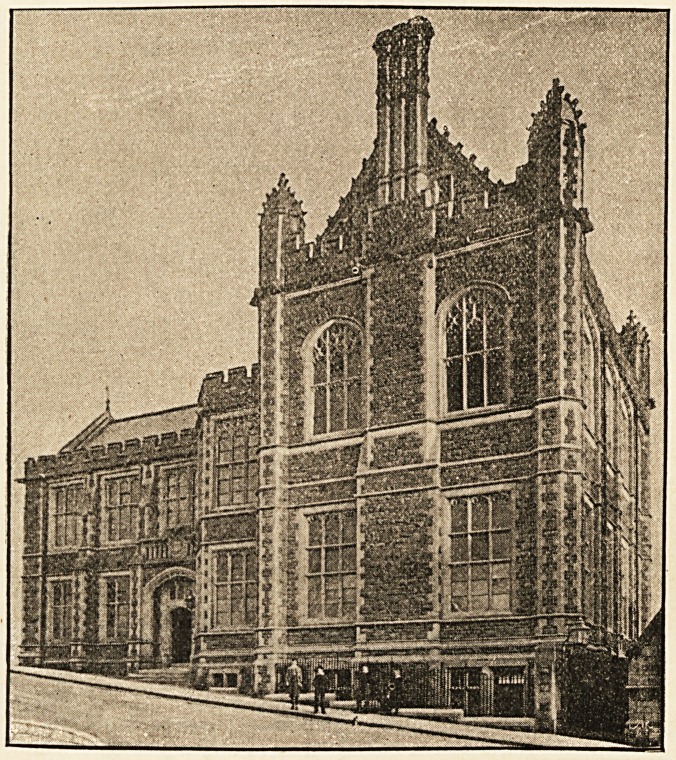


**Figure f4:**
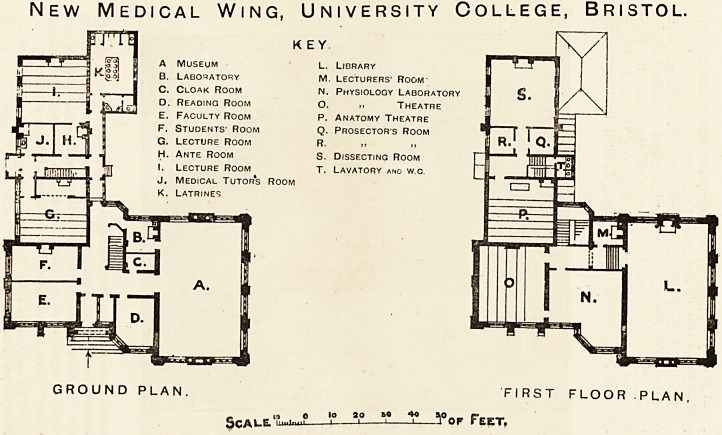


**Figure f5:**
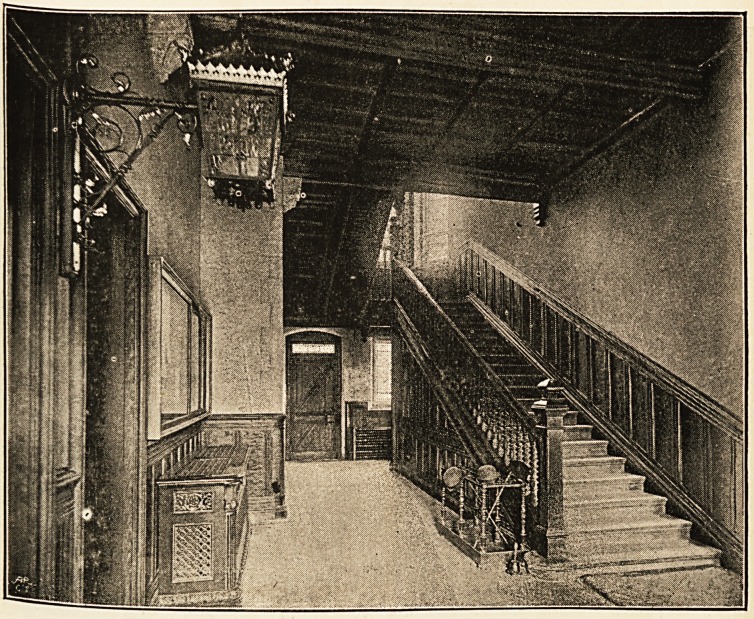


**Figure f6:**
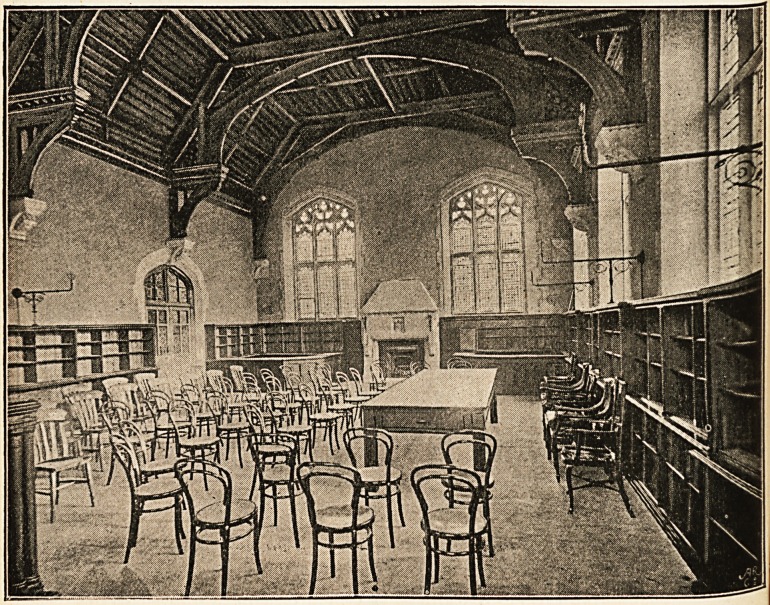


**Figure f7:**
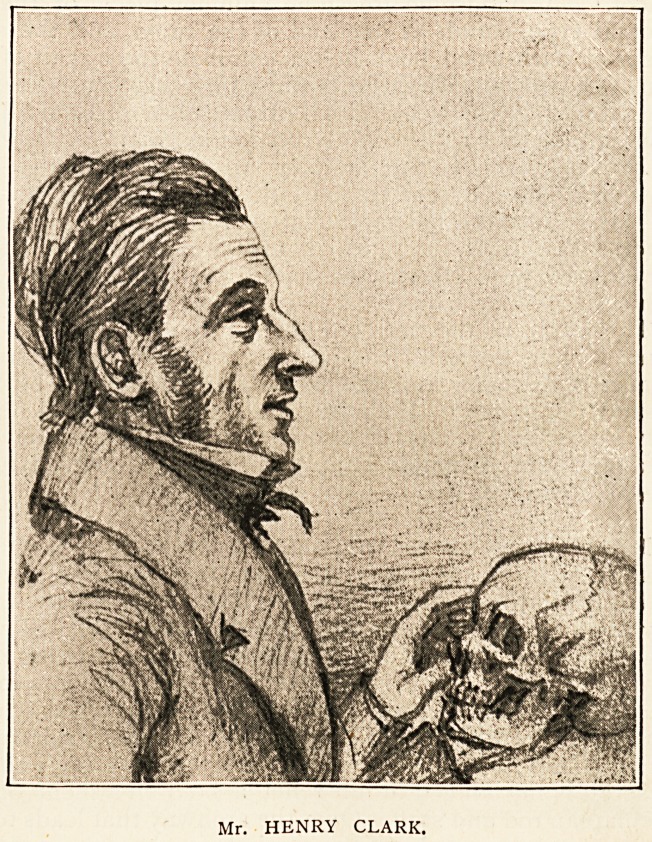


**Figure f8:**
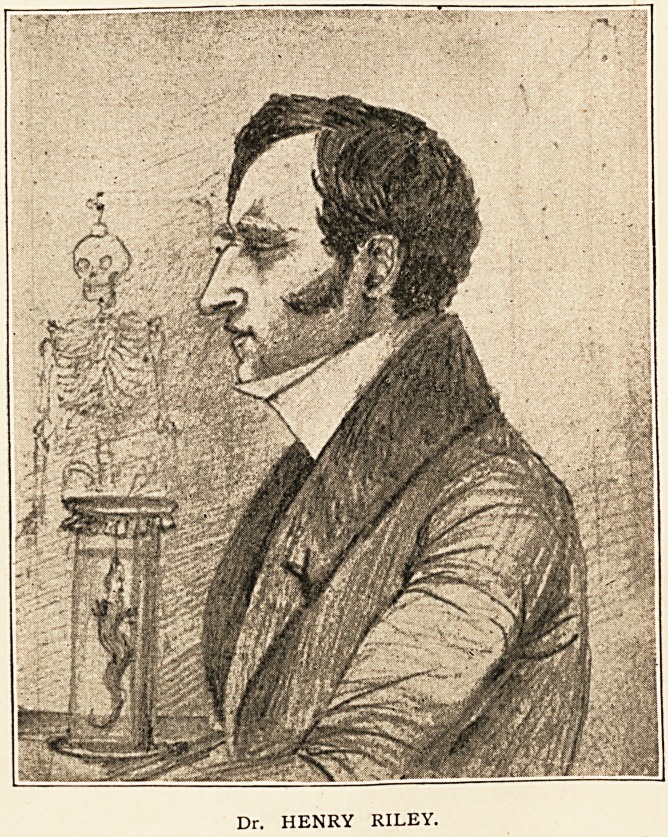


**Figure f9:**
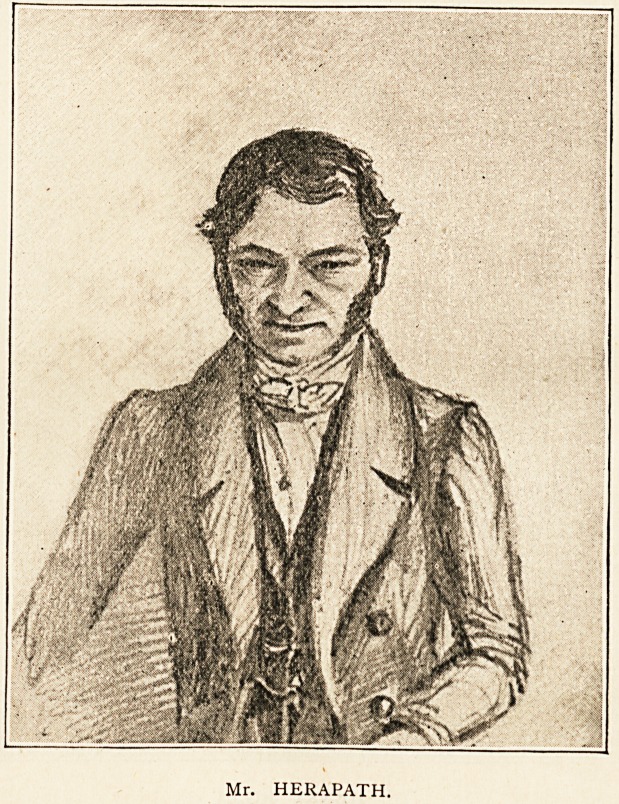


**Figure f10:**
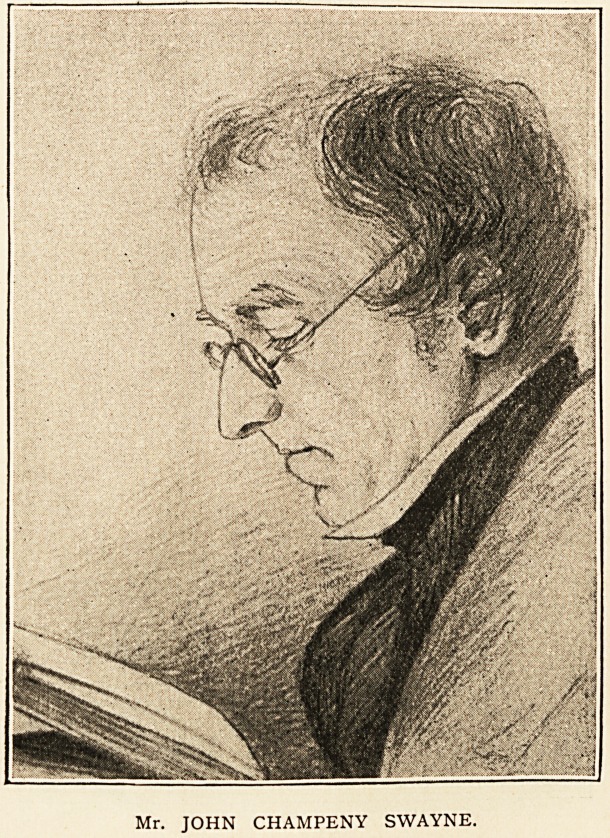


**Figure f11:**
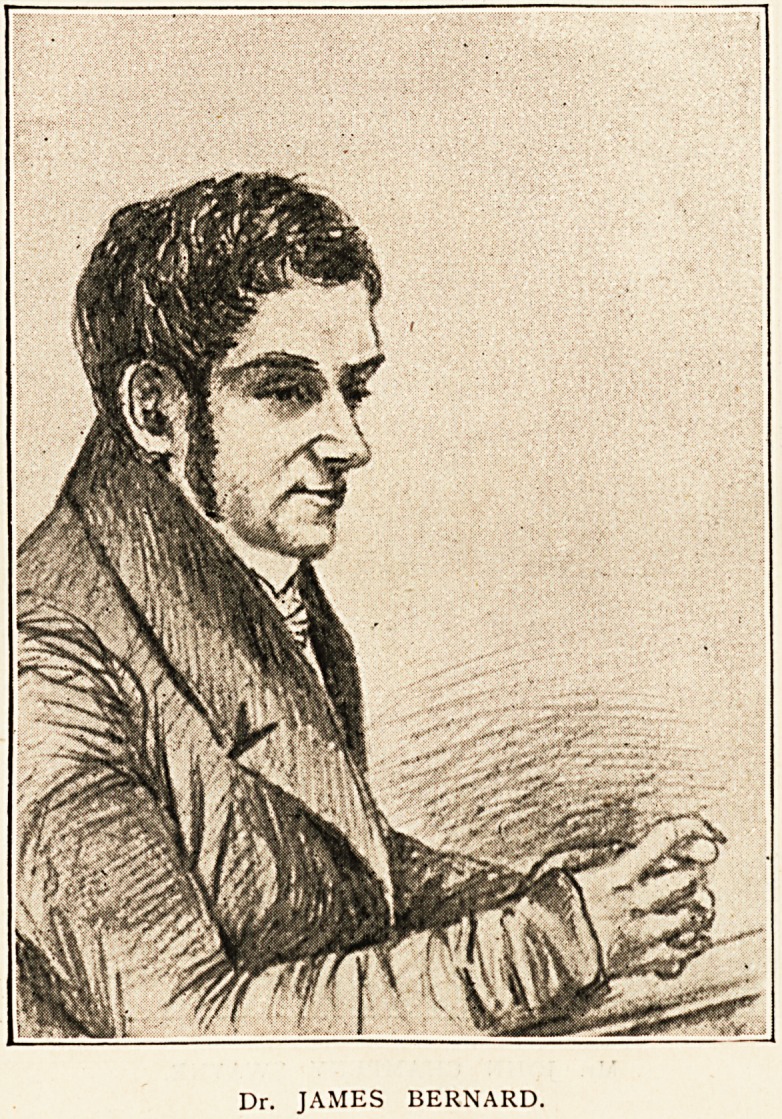


**Figure f12:**